# A Bayesian approach to time-varying latent strengths in pairwise comparisons

**DOI:** 10.1371/journal.pone.0251945

**Published:** 2021-05-20

**Authors:** Blaž Krese, Erik Štrumbelj

**Affiliations:** 1 GEN-I, d.o.o., Ljubljana, Slovenia; 2 Faculty of Computer and Information Science, University of Ljubljana, Ljubljana, Slovenia; Universidad Rey Juan Carlos, SPAIN

## Abstract

The famous Bradley-Terry model for pairwise comparisons is widely used for ranking objects and is often applied to sports data. In this paper we extend the Bradley-Terry model by allowing time-varying latent strengths of compared objects. The time component is modelled with barycentric rational interpolation and Gaussian processes. We also allow for the inclusion of additional information in the form of outcome probabilities. Our models are evaluated and compared on toy data set and real sports data from ATP tennis matches and NBA games. We demonstrated that using Gaussian processes is advantageous compared to barycentric rational interpolation as they are more flexible to model discontinuities and are less sensitive to initial parameters settings. However, all investigated models proved to be robust to over-fitting and perform well with situations of volatile and of constant latent strengths. When using barycentric rational interpolation it has turned out that applying Bayesian approach gives better results than by using MLE. Performance of the models is further improved by incorporating the outcome probabilities.

## Introduction

Modelling pairwise comparisons is an important practical problem and well established in research literature [1, 2]. The foundations were built in the 1950s by Bradley and Terry [3] and Luce [4], though the first idea goes back to Thurstone [5]. The classical approach is the Bradley-Terry model [3]. The model links the pairwise comparison probabilities with the compared objects’ latent strengths, which are in the model’s most simple variant assumed to be constant.

The Bradley-Terry model has been extended in several ways: handling ties [6], ranking individual players in multi-player competitions [7, 8], and stochastic non-transitivity of comparisons [9]. It has also been shown that Bradley-Terry model can be seen as a special case of a more general model. A very recent example of such treatment demonstrates a pairwise comparison model where the Weibull distribution is applied [10]. Another common generalization is to allow for the latent strengths to vary with time and it is the focus of our work. The quintessential application domain for time-varying strength models is sports, where ranking is important both for seeding competitions and for fan engagement. However, a player’s strength changes with age, experience, fatigue, and injuries. And a team’s strength changes with players joining or leaving a team.

The classical time-varying approach is the ELO rating, designed by Arpad Elo [11, 12]. It was adopted, for example, by the International Chess Federation (FIDE) [13] and UEFA [14]. The ELO rating uses a scaled version of the Bradley-Terry model. After each comparison the underlying latent strength is changed with accordance to the previous strength and the output of the comparison. Glickman developed a non-iterative Bayesian algorithm [15]. This model assumes a normal distribution of the latent strengths conditional on the strength at the previous comparison with the standard deviation dependent on the elapsed time between comparisons. Based on this algorithm the Glicko and Glicko-2 rating systems ware developed, where the latter improves on ability to capture sudden changes [16]. One downside of incremental algorithms is that covariance is not taken into account when approximating probability distributions of latent strengths. This was addresses by Coulom [17] who used a Wiener process for the prior of latent strengths and applied it to the Bradley-Terry model, using maximum a posteriori (MAP) inference with Newton’s approximation method. This approach has proven to be better than ELO and Glicko when applied to the game of Go. More recently, Baker and McHale applied deterministic approach to time-varying latent strengths by using barycentric rational interpolation (BRI) [18]. This approach was applied to football where pairwise comparisons were based on the Poisson distribution of the number of goals scored. Baker and McHale also applied BRI to tennis [19], using a symmetric beta distribution for ranking, deduced as a special case of Stern’s gamma model, which can also be reduced to the Bradley-Terry model or Thurstone model. They also showed that BRI outperformed spline interpolation. A model based on the number of goals scored was also used by Owen [20] and Koopman [21], who used an incremental approach to model time dependence of latent strengths with a focus on outcome forecasting rather than hindcasting as in the case of Baker. Cattelan et al. [22] also used an incremental approach to model team’s ability by using an exponentially weighted moving average processes applied to the Bradley-Terry model. Inference was done via maximum likelihood estimation and they applied their model to basketball and football.

In this paper we extend the Bradley-Terry model to allow for time-varying strengths by combining it with barycentric rational interpolants (BRI) [23] or Gaussian processes (GP) [24]. We also extend the model to handle not only binary comparison outcome data but also outcome probabilities, if available to be derived, for example, from bookmakers’ odds. Compared to the majority of related work which is motivated by forecasting, our approach addresses hindcasting. When the focus is on forecasting, the main goal is to minimize the short-term prediction error and for these purpose modelling is based on incremental approach. However, incremental methods are not suitable for hindcasting where it is vital to take into account the covariance between model’s parameters. With hindcasting we are not interested in just the next game output, but rather in the underlying dynamics of latent strengths where a longer period needs to be considered. Research with focus on hindcasting is sparse—Baker and McHale [18, 19] and Coulom [17] who model time-varying latent strengths deterministically with interpolation and the Wiener process, respectively. Compared to Baker and McHale [18, 19] we combined barycentric rational interpolation (BRI) with the Bradley-Terry model and we use Bayesian inference. We also model time-varying strengths with Gaussian processes (GPs). This is similar to Coulom [17], but with two significant differences. First, using GPs is more general, because a Wiener process is a special case of GPs when the kernel function is given by *k*(*t*, *t*′) = min(*t*, *t*′) [25]. And second, we utilize Markov Chain Monte Carlo (MCMC) instead of structural approximation of the posterior and MAP estimation. Notably, our Bayesian models are implemented in Stan [26] and we utilize Markov Chain Monte Carlo for inference. We empirically evaluate and compare the models on toy data and two real-world sports data sets: ATP (Association of Tennis Professionals) tennis and NBA (National Basketball Association) basketball.

## Methodology

### The Bradley-Terry model

Pairwise comparison data are a set of observations, where each observation is the outcome of a pairwise comparison between two objects, where one of the objects is deemed to be superior to the other. We will not consider ties in this paper.

The classical model for such data is the Bradley-Terry model [3] which assumes that the comparison outcome probabilities are governed by unobserved (latent) strengths of the objects. Given a comparison between objects *a* and *b*, we have

$$\begin{array}{c} P\left(a\;\text{is}\;\text{superior}\;\text{to}\;b\right)≜\frac{\theta_{a}}{\theta_{a}+\theta_{b}}, \end{array}$$ (1)

where *θ*_*a*_ and *θ*_*b*_ are the latent strengths of objects *a* and *b*, respectively. In its most basic variant, these strengths are assumed to be constant.

#### Introducing time-varying latent strengths

We will focus on the extensions of the Bradley-Terry model where the latent strengths vary with time. The pairwise comparisons observations are then 4-tuples (*t*_*i*_, *a*_*i*_, *b*_*i*_, *y*_*i*_), where $t_{i}\in R$ is the time when the comparison was made, *a*_*i*_, *b*_*i*_ ∈ {1, …, *K*} are the two objects being compared, from a set of *K* objects, and *y*_*i*_ ∈ {0, 1} is the outcome of the comparison. If object *a*_*i*_ was deemed to be superior to object *b*_*i*_, then *y*_*i*_ = 1, otherwise *y*_*i*_ = 0. Times *t*_*i*_ are not necessarily unique—two comparisons can be made at the same time.

The Bradley-Terry model is a non-deterministic model. The comparison outcome is modeled as a random variable *Y*_*i*_ with support {0, 1}. In general, the probability mass function of *Y*_*i*_ is

$$\begin{array}{c} p\left(y_{i}|\theta ,a_{i},b_{i},t_{i}\right)=P\left(Y_{i}=y_{i}|\theta ,a_{i},b_{i},t_{i}\right), \end{array}$$ (2)

but because *Y*_*i*_ is Bernoulli, we will use the shorthand notation

$$\begin{array}{c} p_{i}≜p\left(1|\theta ,a_{i},b_{i},t_{i}\right), \end{array}$$ (3)

where ***θ*** = ***θ***(*t*) = (*θ*_1_(*t*), …, *θ*_*K*_(*t*)) and *θ*_*j*_(*t*) are the unknown time-dependent latent strengths of the objects.

We can now generalize Eq (1) to

$$\begin{array}{c} p_{i}=\frac{\theta_{a_{i}}\left(t_{i}\right)}{\theta_{a_{i}}\left(t_{i}\right)+\theta_{b_{i}}\left(t_{i}\right)}. \end{array}$$ (4)

In Eq (4) we explicitly write *t*_*i*_ to stress the latent strengths’ dependency on time. To simplify the notation, we will from now on assume this time dependency and omit the times whenever possible.

In order for *p*_*i*_ to be probabilities, the latent strengths have to be positive. Because it is more convenient to work with real parameters *θ*, we typically rewrite Eq (4) as

$$\begin{array}{c} p_{i}=\frac{e^{\theta_{a_{i}}}}{e^{\theta_{a_{i}}}+e^{\theta_{b_{i}}}}=\frac{1}{1+e^{\theta_{b_{i}}-\theta_{a_{i}}}}={\text{logit}}^{-1}\left(\theta_{a_{i}}-\theta_{b_{i}}\right), \end{array}$$ (5)

where logit^−1^ is the cumulative distribution of the standard logistic distribution, also known as the inverse logistic function or inverse logit:

$$\begin{array}{c} {\text{logit}}^{-1}\left(x\right)≜\frac{1}{1+e^{-x}}. \end{array}$$ (6)

This Bradley-Terry model can be viewed as logistic regression with one input variable—the difference between the latent strengths of objects being compared.

#### Model identifiability

Since the outcome probabilities depend only on the difference in latent strengths they are invariant to translation. In order to be able to identify parameters *θ*, we have to set a reference. We set the latent strength of the *K*-th object to be 0 [22].

#### Covariates

In Eq (5) the outcome probability depends solely on the latent strengths of the two objects being compared. In practice, other factors might affect the outcome. For example, home team advantage or weather. We will account for these covariates with a linear term

$$\begin{array}{c} p_{i}={\text{logit}}^{-1}\left(\theta_{a_{i}}-\theta_{b_{i}}+\beta^{⊤}x_{i}\right), \end{array}$$ (7)

where **x**_*i*_ is a vector of covariates for the *i*-th observation and ***β*** is a vector of coefficients. Covariates are assumed to be known and measured without error and coefficients are parameters of the model.

Note that the purpose of this work is not to study the effect that different covariates might have in a particular domain. However, for NBA data we do include a covariate for home team advantage, which is known to have a strong effect on sports match outcome probabilities. The home team advantage covariate *x*_hta,*i*_ can be coded as + 1, −1, or 0 when team *a* is playing at home, team *b* is playing at home, or when the game is played in a neutral venue, respectively.

### Baseline model (BASE)

Our baseline for comparison will be the Bradley-Terry model where we assume that an object’s latent strength is constant ***θ*** = (*θ*_1_, *θ*_2_, …, *θ*_*K*_) and we fit the parameters using maximum likelihood estimation. Given *n* observations, the likelihood is

$$\begin{array}{c} L\left(\theta ,\beta ;y\right)=p\left(y|\theta ,\beta \right)=\prod_{i=1}^{n}p\left(y_{i}|\theta ,\beta \right)=\prod_{i=1}^{n}p_{i}^{y_{i}}{\left(1-p_{i}\right)}^{1-y_{i}}, \end{array}$$ (8)

where the $p_{i}={\text{logit}}^{-1}\left(\theta_{a_{i}}-\theta_{b_{i}}+\beta^{⊤}x_{i}\right)$ as in Eq (7). Then the log-likelihood is

$$\begin{array}{c} \ell \left(\theta ,\beta ;y\right)=\sum_{i=1}^{n}y_{i}\log\left(p_{i}\right)+\left(1-y_{i}\right)\log\left(1-p_{i}\right). \end{array}$$ (9)

Finding the maximum likelihood estimates reduces to the optimization problem

$$\begin{array}{c} \begin{array}{cc} {\hat{\left(\theta ,\beta \right)}}_{\text{BASE}} & =\underset{\left(\theta ,\beta \right)}{\arg\max}\;\ell \left(\theta ,\beta ;y\right)\\ \theta_{K} & =0, \end{array} \end{array}$$ (10)

which we solved using L-BFGS optimization.

### Barycentric rational interpolation model (BRI)

BRI is an alternative to splines. A detailed comparison between BRI and splines is discussed in [27]. BRI is infinitely differentiable, which is a drawback when modelling a process with sudden changes in values. Still, it has been shown that BRI has the same or slightly lower errors in curve fitting than splines. BRI was used to model the attack and defence ability of football teams combined with comparisons of goals scored by the teams modelled with Poisson distribution [18]. A similar study was conducted for ranking tennis players [19].

We start by introducing *m* nodes in time $(t_{k}^{*},\lambda_{k}),\;k=1,2,\ldots ,m$, where λ_*k*_ represents the quantity of interest at time $t_{k}^{*}$. We use the t* notation to make it explicit that these nodes need not correspond to the times of the observations in our data. In practice, we typically use fewer nodes than observations.

The purpose of BRI is to interpolate between these nodes in order to get the quantity of interest at any time. In our case the quantity of interest are unobserved—the latent strengths of objects. We will perform BRI for each object separately. We then write the evolution of the *j*-th object’s latent strength over time in the general barycentric form by interpolation between coordinates [27]

$$\begin{array}{c} \theta_{j}\left(t\right)=\frac{\sum_{k=1}^{m_{j}}w_{jk}\lambda_{jk}/\left(t-t_{jk}^{*}\right)}{\sum_{k=1}^{m_{j}}w_{jk}/\left(t-t_{jk}^{*}\right)}. \end{array}$$ (11)

The number of nodes *m*_*j*_ does not have to be the same for every object, but for our applications we do not lose by assuming that it is. Selecting the number and location of the nodes is analogous to spline interpolation [27]. Domain knowledge can be used but automated optimal placement is infeasible and has to be dealt with heuristically. We positioned the nodes equally spaced in time and empirically selected the best *m* from a finite set of possibilities. As a consequence, the notation $t_{jk}^{*}$ reduces to $t_{k}^{*}$ and weights are given in a simpler form *w*_*jk*_ = (−1)^*k*^, ∀*j* [18].

The general form of the log-likelihood is similar to Eq (9) but **λ** = {λ_*jk*_;} are now the parameters

$$\begin{array}{c} \ell \left(\lambda ,\beta ;y\right)=\sum_{i=1}^{n}y_{i}\log\left(p_{i}\right)+\left(1-y_{i}\right)\log\left(1-p_{i}\right), \end{array}$$ (12)

where $p_{i}={\text{logit}}^{-1}\left(\theta_{a_{i}}-\theta_{b_{i}}+\beta^{⊤}x_{i}\right)$ and

$$\begin{array}{c} \theta_{j}\left(t\right)=\frac{\sum_{k=1}^{m}{\left(-1\right)}^{k}\lambda_{jk}/\left(t-t_{k}^{*}\right)}{\sum_{k=1}^{m}{\left(-1\right)}^{k}/\left(t-t_{k}^{*}\right)},\forall j\neq K. \end{array}$$ (13)

Finding the maximum likelihood estimates reduces to the optimization problem

$$\begin{array}{c} \begin{array}{cc} {\hat{\left(\lambda ,\beta \right)}}_{\text{BRI}} & =\underset{\left(\lambda ,\beta \right)}{\arg\max}\;\ell \left(\lambda ,\beta ;y\right)\\ \theta_{K}\left(t\right) & =0,\forall t, \end{array} \end{array}$$ (14)

which we solved using L-BFGS optimization.

#### Bayesian barycentric rational interpolation model (BRI_bayes_)

We also inferred from the BRI model using the Bayesian framework, treating the **λ** and ***β*** as random variables. The model and prior distributions are

$$\begin{array}{c} \begin{array}{cc} y_{i}|\lambda ,\beta ,t_{i},a_{i},b_{i},x_{i},t^{*} & ∼\text{Bernoulli}({\text{logit}}^{-1}\left(\theta_{a_{i}}-\theta_{b_{i}}+\beta^{⊤}x_{i}\right))\\ \theta_{j}\left(t\right) & =\frac{\sum_{k=1}^{m}{\left(-1\right)}^{k}\lambda_{jk}/\left(t-t_{k}^{*}\right)}{\sum_{k=1}^{m}{\left(-1\right)}^{k}/\left(t-t_{k}^{*}\right)},\forall j\neq K\\ \theta_{K}\left(t\right) & =0,\forall t,\\ \lambda_{j} & ∼N(\mu_{\lambda },\sigma_{\lambda }^{2}\;I),\forall j\neq K\\ \beta & ∼N(0,\;\sigma_{\beta }^{2}\;I). \end{array} \end{array}$$ (15)

It is standard to assume that ***β*** coefficients are centered around 0. The prior constants $\sigma_{\lambda }^{2}$ and $\sigma_{\beta }^{2}$ are user-defined constants. If little or no prior information is available, they can be set to some relatively large value. In the case of ***β*** this value depends on the scale of the covariates. In the case of **λ** this value can be small, because even differences in the order of 10 result in near 1 (or 0) probabilities due to the inverse logit transformation. Note that this model could easily be extended to use regularization on the covariates by placing a hyper-prior on ***β***.

We implemented the model in the Stan probabilistic programming language and inferred from it using the built in variant the No-U-turn Sampler (NUTS), an extension of the Hamiltonian Monte Carlo sampling algorithm [26, 28, 29].

### Gaussian process model (GP)

GPs are a well-studied field with a rich theory [24]. The shape of a GP is determined primarily by its kernel function which is very flexible. By applying different kernel functions we can get for instance a Wiener proces [25] or a certain spline [30]. GPs are also closely connected to some of the more well-known models such as neural networks or support vector machines, but are more intuitive and easy to interpret [24]. On the other hand applying GPs is time demanding due to the covariance matrix inversion which is $O(n^{3})$ where *n* is the number of covariate points [24].

Instead of using BRI we now place a GP prior on each object’s latent strength

$$\theta_{j}\left(t\right)∼\text{GP}\left(m\left(t\right),k\left(t,t^{\prime }\right)\right),\;\forall j,$$

where *m*(*t*) is the mean function and *k*(*t*, *t*′) is the covariance function [24]. The mean function is usually taken to be *m*(*t*) = 0;∀*t*.

The likelihood of the model is the same as in Eq (8), so the posterior distribution is

$$\begin{array}{c} p\left(\theta ,\beta |y\right)\propto L\left(\theta ,\beta ;y\right)\;\prod_{j=1}^{K}\text{GP}\left(0,k\left(t,t^{\prime }\right)\right))=(\prod_{i=1}^{n}p_{i}^{y_{i}}{\left(1-p_{i}\right)}^{1-y_{i}})\;\prod_{j=1}^{K}\text{GP}\left(0,k\left(t,t^{\prime }\right)\right), \end{array}$$ (16)

where $p_{i}={\text{logit}}^{-1}\left(\theta_{a_{i}}-\theta_{b_{i}}+\beta^{⊤}x_{i}\right)$ and we abuse the notation GP to denote the multivariate normal (MVN) probability density function of a GP.

To predict latent strengths θ_*_ ≜ ***θ***(t_*_) for times t_*_, we have to compute the posterior predictive density [31]

$$\begin{array}{c} p\left(\theta_{*},|y\right)=\int p\left(\theta_{*}|\theta \right)\;(\int p(\theta ,\beta |y)\;d\beta )\;d\theta , \end{array}$$ (17)

where *p*(***θ***|**y**) = ∫*p*(***θ***, ***β***|**y**)*d* ***β*** is the marginal posterior obtained by integrating the posterior density over ***β*** (and any kernel hyper-parameters). The conditional multivariate Gaussian distribution *p*(θ_*_|***θ***) is given by

$$\begin{array}{c} \theta_{*}|\theta ∼N\left(K_{t_{*},t}\;K_{t,t}^{-1}\;\theta ,K_{t_{*},t_{*}}-K_{t_{*},t}\;K_{t,t}^{-1}\;K_{t,t_{*}}^{⊤}\right). \end{array}$$ (18)

*K*_⋅,⋅_ are covariance matrices obtained by evaluating kernel functions on different combinations of given times *t* and t_*_.

Eq (17) is only tractable when the likelihood *p*(**y**|***θ***) is normal [31, 32], so no closed form solution exists for our model and we have to resort to numerical methods. One approach is to use structural approximation methods such as Laplace approximation or variational inference, see [24, 31] for a quick overview. For instance, Laplace approximation algorithm uses a quadratic approximation and by optimization locates the mode of the posterior *p*(***θ***|**y**). Variational inference minimizes the divergence between a Gaussian approximation and the posterior distribution, but the likelihood function has to be factored as $p\left(y|\theta \right)=\prod_{i=1}^{n}p\left(y_{i}|\theta_{i}\right)$ [31]. These methods can be quite accurate, especially when the posterior is uni-modal, but they can also give biased results when posterior distribution has a more complex shape. To overcome restrictions of structural approximations we use MCMC sampling algorithms. These methods are more computationally intensive but guarantee convergence in distribution to the posterior in the limit of long runs [31].

The model and prior distributions are governed by

$$\begin{array}{c} \begin{array}{cc} y_{i}|\theta ,\beta ,t_{i},a_{i},b_{i},x_{i} & ∼\text{Bernoulli}(p_{i})\\ p_{i} & ={\text{logit}}^{-1}\left(\theta_{a_{i}}-\theta_{b_{i}}+\beta^{⊤}x_{i}\right)\\ \theta_{j}|\ell ,\sigma & ∼\text{MVN}(0,K\left(t,t^{\prime }|\sigma ,\ell \right)),\;\forall j\neq K\\ \theta_{K}\left(t\right) & =0,\;\forall t\\ \sigma & ∼N(0,\;\sigma_{\sigma }^{2}),\;\sigma >0\\ \ell & ∼\text{GIG}(a_{\text{gig}},b_{\text{gig}},q_{\text{gig}})\\ \beta & ∼N(0,\;\sigma_{\beta }^{2}\;I). \end{array} \end{array}$$ (19)

The choice of prior distributions requires additional explanation. For the kernel function *k*(*t*, *t*′|*σ*, *ℓ*) we considered the most commonly used squared exponential kernel

$$\begin{array}{c} k\left(r\right)=\sigma^{2}\;\text{exp}(\frac{-r^{2}}{2\ell^{2}}), \end{array}$$ (20)

where *r* = |*t* − *t*′|, and three Matérn kernels

$$\begin{array}{c} \begin{array}{cc} k_{\nu =\frac{1}{2}}\left(r\right) & =\sigma^{2}\;\text{exp}(-\frac{r}{\ell }),\\ k_{\nu =\frac{3}{2}}\left(r\right) & =\sigma^{2}\;(1+\frac{\sqrt{3}r}{\ell })\;\text{exp}(-\frac{\sqrt{3}r}{\ell }),\\ k_{\nu =\frac{5}{2}}\left(r\right) & =\sigma^{2}\;(1+\frac{\sqrt{5}r}{\ell }+\frac{5r^{2}}{3\ell^{2}})\;\text{exp}(-\frac{\sqrt{5}r}{\ell }). \end{array} \end{array}$$ (21)

Note that lim_*ν* → ∞_ *k*_*ν*_(*r*) = *k*(*r*). Each kernel also has hyper-parameters that need to be properly chosen, that are deviation *σ* and length-scale *ℓ*. For *σ* we have set prior mean to 0, but only consider positive non-zero values. This choice is due to the fact that latent strength can either be close to constant corresponding to stagnation or very wavy when some significant changes occur.

**Fig 1:**
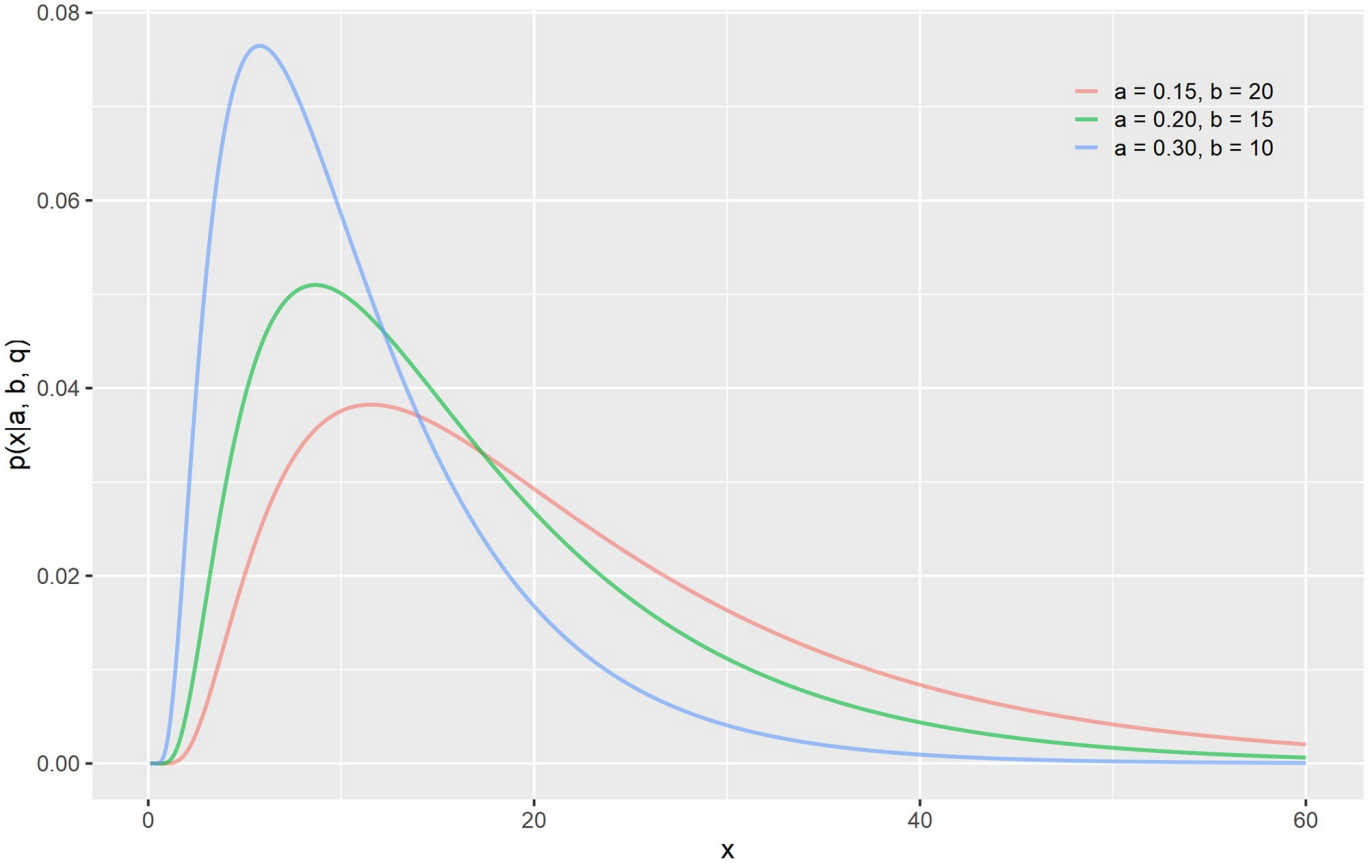
GIG probability density function. The GIG probability density function with *q* = 1 and different values of *a* and *b*.

We put a generalized inverse Gaussian (GIG) prior on the length-scale *ℓ* estimation. The GIG probability density function is given by

$$\begin{array}{c} p\left(x\;|\;a,b,q\right)=\frac{{\left(\frac{a}{b}\right)}^{\frac{q}{2}}}{2K_{q}\left(\sqrt{ab}\right)}x^{q-1}\;\text{exp}(-\frac{1}{2}(ax+\frac{b}{x})), \end{array}$$ (22)

where $x,a,b\in R^{+}$, q∈Z and *K*_*q*_ represents a modified Bessel function of second kind. We chose the GIG distribution, because it has a sharp left tail putting very little probability mass on close-to-zero length-scales. The right-hand side the GIG has a thin tail which allows us to keep out the very large length-scales. We set *q*_gig_ = 1 and determined *a*_gig_ and *b*_gig_ by optimization such that the mode of the GIG was equal to the distance between time nodes (see subsection Auxiliary nodes for more efficient computation). Fig 1 shows how the parameters *a*_gig_ and *b*_gig_ allow for enough flexibility for our purposes even when keeping *q*_gig_ fixed to 1.

#### Gaussian process model with outcome probabilities (GP_prob_)

Sometimes additional data are available in the form of probabilistic predictions ${\hat{p}}_{i}$, which estimate the unknown outcome probabilities *p*_*i*_. For example, probabilities derived from odds in sports, which are known to be good estimates of outcome probabilities [33].

Probabilistic predictions, even if moderately biased, should provide more information than binary outcomes. We extend the model from Eq (19) to allow for the inclusion of such data:

$$\begin{array}{c} \begin{array}{cc} {\hat{p}}_{i}|\tau & ∼\text{Beta}(p_{i}\;\tau ,\left(1-p_{i}\right)\;\tau )\\ \tau & ∼\text{Uniform}(0,\tau_{\text{max}}). \end{array} \end{array}$$ (23)

We assume that the probability estimates are beta-distributed with the mean equal to the unknown true probability. The hyper-parameter *τ* can be interpreted as the quality of the source of probability estimates—smaller values indicate better probabilities.

#### Auxiliary nodes for more efficient computation

In certain domains, for example, in most professional sports, the comparisons are few and far apart and a single comparison provides very little information about the latent strengths, so we need a relatively long period of time to get a good estimate of latent strength. In the context of GPs, we can deal with this by increasing the length-scale. However, a larger length-scale results in more correlation in the posterior and therefore less efficient exploration of the posterior via MCMC.

To allow for more efficient computation, we introduce auxiliary nodes (time points), similar to BRI. The likelihood is computed only at these nodes and each observation is assigned to the nearest auxiliary node. In the extreme case where an auxiliary node is placed at each observation, the method reduces to the initially described model.

## Empirical evaluation

We empirically evaluated and compared the models on three data sets: a toy data set and two real world data sets: ATP (Association of Tennis Professionals) and NBA (National Basketball Association). We collected ATP data for the 20 players with the most games in the 5 seasons in the period from 2015 to 2019, for a total of 673 matches. We collected NBA game outcomes for 5904 regular season games in the 5 seasons period from 2013 to 2018. For the NBA data we also obtained bookmakers’ wining odds for every match in the selected seasons period. The resources for data are the following:

- ATP: https://datahub.io/sports-data/atp-world-tour-tennis-data
- NBA: https://www.basketball-reference.com/
- NBA odds: https://www.betexplorer.com/

The raw data are available as S1–S4 Datasets.

### Toy data

In the toy data set we compare 3 objects. The main feature of the data is a discontinuity in the latent strengths of the first and the second object. The latent strengths are:

$$\begin{array}{c} \begin{array}{cc} \theta_{1}\left(t\right) & =-2\;H(t-250)+1;\\ \theta_{2}\left(t\right) & =2\;H(t-167)-1;\\ \theta_{3}\left(t\right) & =0, \end{array} \end{array}$$ (24)

where H(⋅) stands for the Heaviside function and *t* ∈ {0, 1, 2, …, 499}.

The 3rd object’s latent strength is held at constant value of 0. For the 1st object latent strength *θ*_1_(*t*) is constant at value 1 for times 0 ≤ *t* ≤ 250 and then jumps to value −1 for 250 < *t* < 500. The shape for the 2nd object is complementary, i.e. *θ*_2_(*t*) jumps from value −1 to 1 at time 167. The difference in latent strengths of value 1 corresponds to approximately a 73% chance of winning for the object with the higher latent strength.

In order to simulate comparison data we need to determine which objects are to be compared. Given three objects there are 3 possible combinations of pairwise comparisons. Each of the combinations was selected with a 50% probability for each time point *t*_*i*_ ∈ *t*. Win probabilities *p*_*i*_ are given with Eq (5) and the outputs of comparisons are determined with a sample from *y*_*i*_|*p*_*i*_ ∼ Bernoulli(*p*_*i*_).

#### Model evaluation and parameter tuning

We evaluated the models using the log-score and train-test (holdout) estimation repeated 10 times to account for train-test split variability. We approximated the standard error of the estimates using hierarchical bootstrap, accounting for inter-observation and inter-train-test split variability.

The models have several tunable parameters. For every experiment and every train-test split separately, their values were selected before training the model from a predetermined set of candidate values using internal train-test estimation on the training set, repeated 5 times.

A summary of experiments’ settings for each data set is in Table 1. For the ATP and NBA data set we used half of the data for training. For the toy data set we used only 10% of data for the training—because these data are simulated, we could generate as many training observations as necessary to reduce the standard errors of the log-score estimates. For all three data sets we used a 90%-10% train-test split for internal selection of parameters.

**Table 1:** Experiments’ parameters settings.

	Toy	ATP	NBA
Train data ratio [%]	10	50	50
Internal train data ratio [%]	90	90	90
#train-test splits	10	10	10
#internal train-test splits	5	5	5
Models	**BASE**, **BRI**, **BRI**_bayes_, **GP**, **GP**_prob_	**BASE**, **BRI**, **BRI**_bayes_, **GP**,	**BASE**, **BRI**, **BRI**_bayes_, **GP**, **GP**_prob_
#nodes	(1, 2, 3, 5, 10, 15, 20)	(1, 5, 10, 20, 30, 50)	(1, 5, 10, 20, 30, 50)
Kernels *k*_*ν*_	$\nu \in \{\frac{1}{2},\frac{3}{2},\frac{5}{2},\infty \}$	$\nu \in \{\frac{1}{2},\frac{3}{2},\frac{5}{2},\infty \}$	$\nu \in \{\frac{1}{2},\frac{3}{2},\frac{5}{2},\infty \}$
Prior parameters $\left(\mu_{\lambda },\sigma_{\lambda }^{2},\sigma_{\beta }^{2},\sigma_{\sigma }^{2}\right)$	(**0**, 4, 1, 1)	(**0**, 4, 1, 1)	(**0**, 4, 1, 1)

Experiment settings and candidate tunable parameter values.

We did not use the **GP**_prob_ model on the ATP data, because the data do not include outcome probabilities. For toy data we used a different set of nodes than with ATP and NBA data due to different time spans.

In the priors we set ***μ***_λ_ = **0** since the reference object with *θ*_*K*_(*t*) = 0, ∀*t* was selected randomly with no prior knowledge on relation to other objects’ latent strengths. The corresponding variance was set to $\sigma_{\lambda }^{2}=4$. This is based on the assumption that teams in a competition are homogeneous in strength. It roughly corresponds to that a bottom 25% team has at least a 10% chance to beat a top 25% team. The variance hyper-parameter $\sigma_{\beta }^{2}$ for the home advantage prior was set to 1, which corresponds to ≈27% of increase in win probability. The same value was set to $\sigma_{\sigma }^{2}$ for the kernels’ hyper-parameter *σ* which gives our prior belief on the rate of variation of the latent strength. For the Bayesian models we used 200 warmup and 800 sampling iterations. Effective sample sizes and R-hat diagnostics did not indicate any issues with MCMC. For the **GP**_prob_ model the hyper-parameter *τ*_*max*_ was set to 1000.

### Results

#### Tunable parameter values

The selected tunable parameters for each train-test split are shown in S1–S3 Tables in S1 Appendix, for toy, ATP, and NBA data sets, respectively:

- **Toy**: The parameters vary a lot between train-test splits. This is expected since there are discontinuities in the latent strengths and only 10% of the data were used for training. The two BRI-based models are similar as are the two **GP** models—any differences are difficult to discern due to the high variability. For the **GP**_prob_ model the number of nodes is mostly larger than with other models. Additional information in the form of probabilities allows for a smaller length-scale and a more detailed curve.
- **ATP**: A single node is consistently selected for both BRI-based models with a single exception in case of **BRI**_bayes_. The number of selected nodes for the **GP** model varies more, but 1 and 5 nodes are the most common, also suggesting a larger length-scale and that the models do not find a lot of variability in players’ latent strengths.
- **NBA**: The number of nodes for the BRI-based models varies from 1 to 5 and the number of nodes for the **GP** model varies from 5 to 20. This suggests that NBA data has more variability in latent strengths than ATP data. For the **GP**_prob_ model the maximum allowed number of nodes (50) is consistently selected with only one exception where 30 nodes is selected. Additional information in the form of probabilities allows for a smaller length-scale and a more detailed curve. This also suggests that our estimate of the model performance is biased (pessimistic)—allowing a larger number of nodes could lead to even better performance.

#### Model performance

We organized the model performance results into upper-triangular tables where each row and column correspond to one of the models. Above-diagonal elements are the mean log-score differences between the row and column models. These elements facilitate a direct comparison of the two models. Diagonal elements are the estimated log-scores for a particular model. The results on toy data set are in Table 2.

**Table 2:** Model performance on toy data set.

	**BASE**	**BRI**	**BRI**_bayes_	**GP**	**GP**_prob_
**BASE**	−0.709±0.006	**−** **0.040** **±** **0.033**	**−** **0.105** **±** **0.017**	**−** **0.122** **±** **0.012**	**−** **0.176** **±** **0.011**
**BRI**		−0.669±0.032	**−** **0.066** **±** **0.029**	**−** **0.082** **±** **0.026**	**−** **0.136** **±** **0.032**
**BRI**_bayes_			−0.603±0.014	**−** **0.017** **±** **0.010**	**−** **0.071** **±** **0.011**
**GP**				−0.587±0.009	**−** **0.054** **±** **0.009**
**GP**_prob_					−0.533±0.008

Diagonal elements are the estimated log-scores. Above-diagonal elements are the estimated difference between the log-scores of the corresponding row and column models. Standard errors of the estimates are provided and differences greater than 1 standard error are in bold.

**Fig 2:**
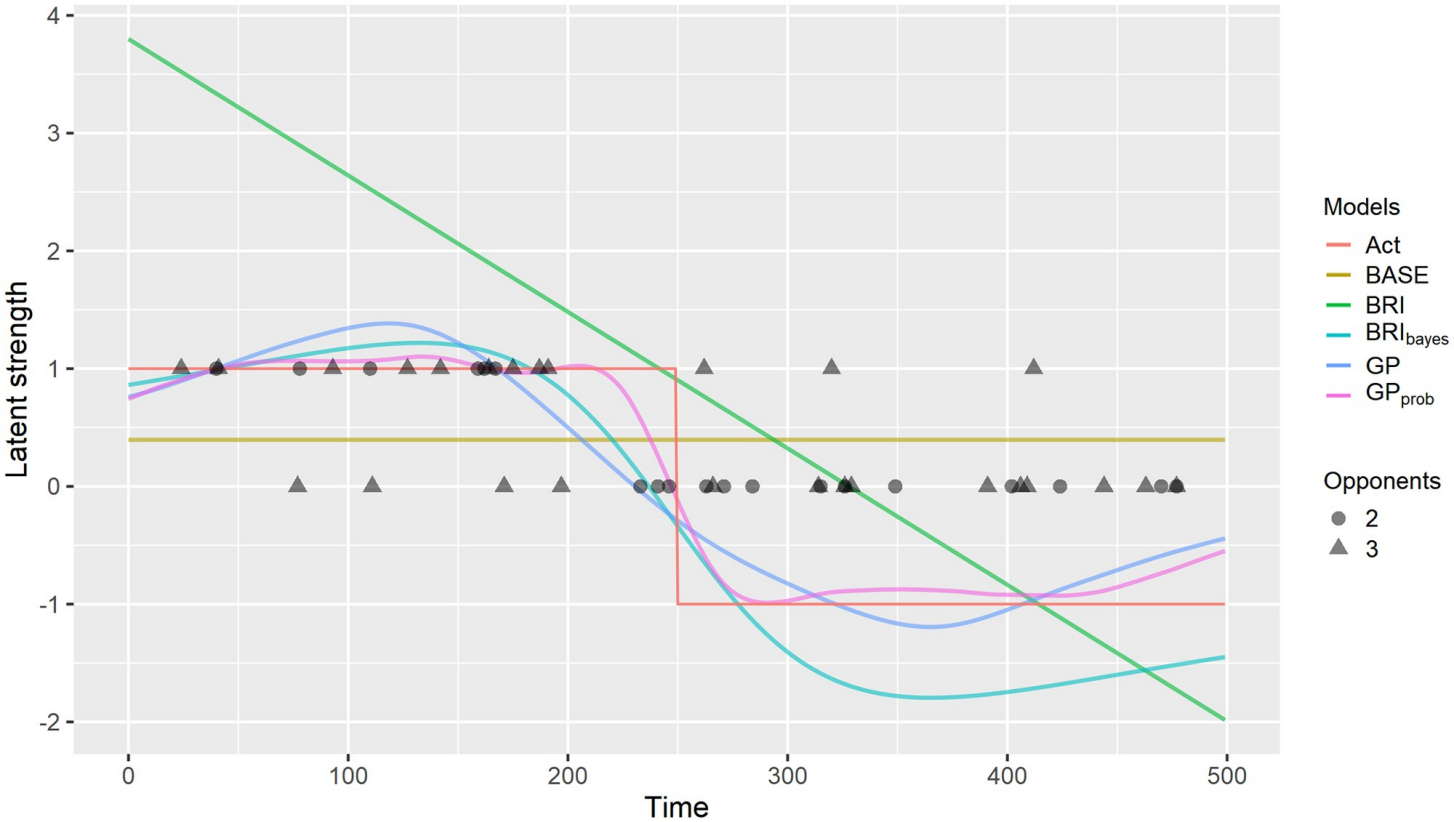
Model comparison of estimated latent strength for the 1st object in the toy data set. For models **BRI**_bayes_, **GP** and **GP**_prob_ we show the posterior mean. The red line represents the true latent strength. The points represent the training data. **GP** fits the true latent strength better than **BRI**_bayes_. **GP**_prob_, which uses additional probability data fits the true latent strength best.

All the models outperform the benchmark model **BASE**. In increasing order of performance, the models are **BASE**, **BRI**, **BRI**_bayes_, **GP**, and **GP**_prob_. The latter was expected to outperform the other models, because it uses more information. **GP** is better than the BRI-based models at handling the discontinuity in the latent strength. Fig 2 shows an illustrative example.

We note that in this particular illustration 2 nodes were selected for the **BRI** model and thus a linear solution, while for **BRI**_bayes_ and **GP** models 3 nodes were selected resulting in solutions with a closer fit.

The results on ATP data are in Table 3. As the selected parameters already suggested, the models find no meaningful variability in latent strengths and none of the models outperform the baseline model **BASE**, which assumes constant latent strengths. This can either be due to the top players indeed being consistent throughout the observed period or due to lack of information. Additional information could be incorporated, such as matches with players outside the top players and court-type, which plays an important role. However, this example illustrates that the more flexible models are robust to over-fitting the data and do not perform worse than a constant latent-strength model. We also note that in case of the **BRI** model only one node was chosen for all train-test splits giving the same result as the **BASE** model.

**Table 3:** Model performance on ATP data set.

	**BASE**	**BRI**	**BRI**_bayes_	**GP**
**BASE**	−0.581±0.014	−0.000±0.000	−0.000±0.006	0.008±0.010
**BRI**		−0.581±0.014	−0.000±0.006	0.008±0.010
**BRI**_bayes_			−0.580±0.013	**0.008** **±** **0.006**
**GP**				−0.589±0.011

Diagonal elements are the estimated log-scores. Above-diagonal elements are the estimated difference between the log-scores of the corresponding row and column models. Standard errors of the estimates are provided and differences greater than 1 standard error are in bold.

**Fig 3:**
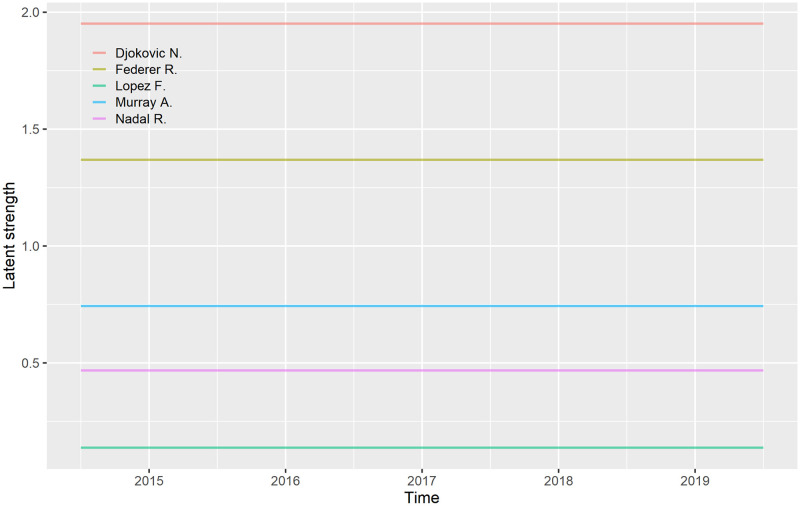
The five players with the highest latent strength according to the BASE model.

In Fig 3 we show latent strengths of top 5 tennis players obtained with the **BASE** model. These results show that from 2015 to 2019 Novak Djoković was the best player followed by Roger Federer, Andy Murray, Rafael Nadal, and Feliciano Lopez.

**Fig 4:**
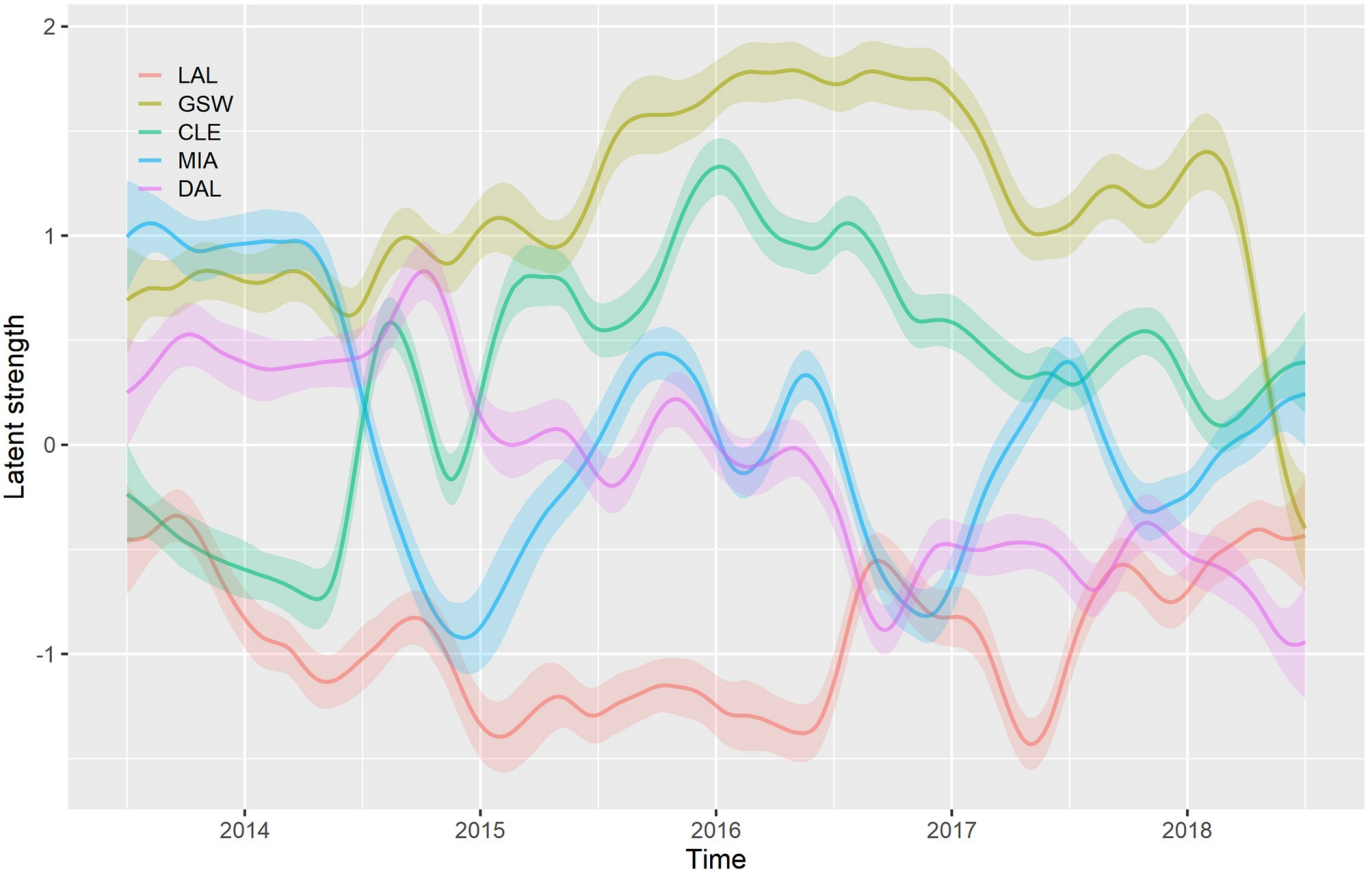
Comparison of latent strengths of selected five NBA teams using the GP_prob_ model. For each team a line and a ribbon are shown which represent a posterior mean and the corresponding standard deviation. The Golden States Warriors (GSW) were for most of the period the best out of these five teams. A drop can be seen in Miami Heat’s (MIA) strength going from the 2014 to the 2015 season, while the Cleveland Cavaliers’s (CLE) strength increases. These changes correspond with LeBron James leaving Miami Heat and returning to Cleveland Cavaliers.

The results on NBA data are in Table 4. Unlike ATP data set, the selected tunable parameter values suggested that there is some variability in latent strengths to be modelled. Similar to toy data set the models are, in order of increasing performance, **BASE**, **BRI**, **BRI**_bayes_, **GP**, and **GP**_prob_. Again, the **GP**_prob_ model was expected to outperform the other models, because it uses more information and the **GP** model is better than the BRI-based models. As an additional benchmark we include a comparison with probabilities from bookmaker win odds (**Odds**). Our model when using these probabilities outperforms them. The other models give 3%—6% lower log-scores. The latent strengths of 5 selected NBA teams are shown in Fig 4.

**Table 4:** Model performance on NBA data set.

	**BASE**	**BRI**	**BRI**_bayes_	**GP**	**GP**_prob_	**Odds**
**BASE**	−0.627±0.002	**−** **0.005** **±** **0.002**	**−** **0.009** **±** **0.003**	**−** **0.022** **±** **0.002**	**−** **0.047** **±** **0.002**	**−** **0.042** **±** **0.002**
**BRI**		−0.622±0.003	**−** **0.004** **±** **0.002**	**−** **0.017** **±** **0.002**	**−** **0.042** **±** **0.003**	**−** **0.037** **±** **0.003**
**BRI**_bayes_			−0.618±0.004	**−** **0.013** **±** **0.003**	**−** **0.038** **±** **0.003**	**−** **0.033** **±** **0.003**
**GP**				−0.605±0.002	**−** **0.025** **±** **0.002**	**−** **0.019** **±** **0.002**
**GP**_prob_					−0.580±0.003	**0.005** **±** **0.001**
**Odds**						−0.586±0.003

Diagonal elements are the estimated log-scores. Above-diagonal elements are the estimated difference between the log-scores of the corresponding row and column models. Standard errors of the estimates are provided and differences greater than 1 standard error are in bold.

## Conclusions

In this paper we extended the Bradley-Terry model using BRI and GPs to model latent strengths as the time-varying components of the model. In addition the model also allows for the inclusion of covariates and outcome probabilities. The use of outcome probabilities is overlooked in related work, although they are often available and substantially improve the model’s performance as we demonstrated on toy and real data from NBA games. Even a biased estimate of the outcome probability provides more information than observing a single realization of the process.

We empirically demonstrated the advantages of GPs over BRI and the benefits of using a Bayesian approach to BRI instead of MLE. The BRI-based models are more sensitive to node selection than the GP-based models, the Bayesian BRI model less so than the MLE-based model. All the investigated models are robust to over-fitting and perform well even when the latent strengths are constant. As expected, BRI does not handle discontinuities as well as GPs. However, it is worth noting that this issue is not as pronounced when modelling latent strengths in a log-odds setting as it is when modelling observed data. Due to the exponential transformation, relatively sharp changes in observed performance can be modelled well by a smoother change in latent strength. This is an argument in favour of BRI as a useful alternative to splines and GPs when modelling latent strengths.

In our research we focused on hindcasting rather than forecasting. That is why we evaluated our models based on their performance on left-out games. If the goal was forecasting, we acknowledge that other approaches tailored to forecasting would give better results. Note, however, that our **GP**_prob_ model gives better results than log-scores calculated form bookmakers’ odds. The down-side of our approach is the time complexity which comes with the MCMC methods and calculations of covariance matrix inverses. On the other hand our results are valuable to get a quantitative insight about the underlying strength dynamics of players or teams, which can be used for seeding competitions and matchmaking, scouting or visually engaging coaches and fans.

We could further improve our models in two ways. One direction is to use some other probability distribution function for modelling the comparison outcome which might be more suited to specific data. Another upgrade of the model would be to incorporate transitivity effect, which is often present in sports data.

## Supporting information

S1 Appendix (PDF)

S1 Dataset ATP data. (CSV)

S2 Dataset NBA games data. (CSV)

S3 Dataset NBA win odds data. (CSV)

S4 Dataset NBA teams data. (CSV)
