# Supplementary material for: A Bayesian approach to time-varying latent strengths in pairwise comparisons
Source: PLoS One. 2021 May 20;16(5):e0251945. doi: 10.1371/journal.pone.0251945 (PMC8136743; doi:10.1371/journal.pone.0251945)
Supplement: S1 Appendix — (PDF) [file pone.0251945.s001.pdf]

S1 Appendix

S1 Table. Selected tunable parameter values for each train-test split of toy data set.

	**BASE**	**BRI**	**BRI$_{\text{bayes}}$**	**GP**	**GP$_{\text{prob}}$**
1	$(/, /)$	$(10, /)$	$(10, /)$	$(5, \nu = 5/2)$	$(15, \nu = 3/2)$
2	$(/, /)$	$(2, /)$	$(3, /)$	$(5, \nu = \infty)$	$(15, \nu = \infty)$
3	$(/, /)$	$(1, /)$	$(20, /)$	$(2, \nu = \infty)$	$(20, \nu = 3/2)$
4	$(/, /)$	$(3, /)$	$(3, /)$	$(10, \nu = \infty)$	$(20, \nu = 3/2)$
5	$(/, /)$	$(1, /)$	$(1, /)$	$(10, \nu = 1/2)$	$(2, \nu = \infty)$
6	$(/, /)$	$(2, /)$	$(10, /)$	$(15, \nu = 5/2)$	$(10, \nu = 5/2)$
7	$(/, /)$	$(3, /)$	$(3, /)$	$(5, \nu = \infty)$	$(5, \nu = \infty)$
8	$(/, /)$	$(3, /)$	$(3, /)$	$(15, \nu = \infty)$	$(20, \nu = 3/2)$
9	$(/, /)$	$(2, /)$	$(3, /)$	$(3, \nu = \infty)$	$(10, \nu = 3/2)$
10	$(/, /)$	$(3, /)$	$(5, /)$	$(2, \nu = 5/2)$	$(2, \nu = \infty)$

S2 Table. Selected tunable parameter values for each train-test split of ATP data set.

	BASE	BRI	$\text{BRI}_{\text{bayes}}$	GP
1	$(/,/)$	$(1,/)$	$(1,/)$	$(5,\nu=\infty)$
2	$(/,/)$	$(1,/)$	$(1,/)$	$(1,\nu=\infty)$
3	$(/,/)$	$(1,/)$	$(1,/)$	$(5,\nu=3/2)$
4	$(/,/)$	$(1,/)$	$(5,/)$	$(20,\nu=5/2)$
5	$(/,/)$	$(1,/)$	$(1,/)$	$(10,\nu=5/2)$
6	$(/,/)$	$(1,/)$	$(1,/)$	$(10,\nu=1/2)$
7	$(/,/)$	$(1,/)$	$(1,/)$	$(1,\nu=5/2)$
8	$(/,/)$	$(1,/)$	$(1,/)$	$(5,\nu=5/2)$
9	$(/,/)$	$(1,/)$	$(1,/)$	$(1,\nu=\infty)$
10	$(/,/)$	$(1,/)$	$(1,/)$	$(5,\nu=5/2)$

S3 Table. Selected tunable parameter values for each train-test split of NBA data set.

	BASE	BRI	$\text{BRI}_{\text{bayes}}$	GP	GP_{prob}
1	$(/,/)$	$(1,/)$	$(1,/)$	$(5,\nu=1/2)$	$(50,\nu=1/2)$
2	$(/,/)$	$(5,/)$	$(5,/)$	$(20,\nu=1/2)$	$(50,\nu=1/2)$
3	$(/,/)$	$(1,/)$	$(1,/)$	$(20,\nu=3/2)$	$(50,\nu=1/2)$
4	$(/,/)$	$(1,/)$	$(1,/)$	$(5,\nu=1/2)$	$(50,\nu=3/2)$
5	$(/,/)$	$(5,/)$	$(5,/)$	$(30,\nu=1/2)$	$(50,\nu=1/2)$
6	$(/,/)$	$(1,/)$	$(5,/)$	$(20,\nu=1/2)$	$(50,\nu=1/2)$
7	$(/,/)$	$(5,/)$	$(5,/)$	$(20,\nu=1/2)$	$(50,\nu=1/2)$
8	$(/,/)$	$(5,/)$	$(5,/)$	$(10,\nu=5/2)$	$(30,\nu=3/2)$
9	$(/,/)$	$(1,/)$	$(5,/)$	$(5,\nu=\infty)$	$(50,\nu=1/2)$
10	$(/,/)$	$(5,/)$	$(5,/)$	$(5,\nu=3/2)$	$(50,\nu=3/2)$
